# Optimal Sensor Placement via a POD-QR Framework for High-Fidelity 3D Temperature Field Reconstruction in Large-Scale Ultra-Low Temperature Chest Freezers

**DOI:** 10.3390/s26082441

**Published:** 2026-04-16

**Authors:** Yisha Chen, Jianguo Qu, Yunfeng Xue, Baolin Liu, Jiecheng Tang, Jianxin Wang

**Affiliations:** 1 Shanghai Origincell Biological Cryo Equipment Co., Ltd., 380 Quyou Rd., Shanghai 201399, China; jianguo@origincell.com (J.Q.); xueyunfeng@origincell.com (Y.X.); curtis.tang@hotmail.com (J.T.); 2 School of Health Science and Engineering, University of Shanghai for Science and Technology, 516 Jungong Rd., Shanghai 200093, China; blliuk@163.com

**Keywords:** temperature field reconstruction, principal component analysis, optimal sensor placement, sensing network, low-temperature freezer

## Abstract

Reliable temperature distribution measurement in ultra-low temperature (ULT) chest freezers is crucial for preserving biospecimen integrity in cryopreservation, but dense sensor arrays required for accuracy are often impractical due to space constraints and cost limitations. To address this critical challenge, this work presents a systematic data-driven framework for optimal sensor placement in large-scale (3 m^3^) ULT chest freezers under stable operating conditions. To our knowledge, it is the first realization of high-fidelity cryogenic temperature field reconstruction coupled with sparse sensor layout optimization tailored to large-volume ULT chest freezers. First, high-resolution reference temperature fields were constructed via universal kriging interpolation, validated with leave-one-out cross-validation (LOOCV) to achieve mean absolute error (MAE) ≤0.67 °C and coefficient of determination $R^{2}>0.92$. Principal component analysis (PCA) was then applied to training data to extract a tailored proper orthogonal decomposition (POD) basis. The first three principal components captured 99.8% of cumulative energy. Optimal sensor locations were determined via QR-column pivoting on the rank-3 POD basis, converging to a minimal configuration of 3 sensors (a 94% reduction from the 48-sensor full-scale setup). This sparse sensor network achieved exceptional reconstruction performance: grid-level MAE ≤0.079 °C and root mean squared error (RMSE) ≤0.093 °C against reference fields ($R^{2}\geq 0.999$), while point-level validation against experimental measurements yielded MAE ≤0.502 °C and RMSE ≤0.842 °C ($R^{2}\geq 0.971$). The results demonstrate that, for large-scale ULT chest freezers, the proposed data-driven approach is capable of automatically determining an optimal sparse sensor subset and enabling reliable 3D cryogenic temperature field reconstruction for efficient thermal monitoring. By resolving the trade-off between monitoring accuracy, space efficiency, and cost-effectiveness, this framework provides a scientifically rigorous alternative to empirical sensor deployment standards, offering practical scalability for cryogenic biobanking applications.

## 1. Introduction

Ultra-low temperature (ULT) freezers are indispensable core equipment for long-term cryopreservation of biological specimens, including cell cultures, blood products, genomic DNA, proteins, and vaccines, forming the foundation of biobanking, clinical research, and global public health initiatives [1]. However, thermal instability and temperature heterogeneity within ULT freezer chambers pose critical threat to sample integrity. Existing studies have shown that temperature fluctuations can degrade cell recovery rates and impair T-cell functional activity [2]. To mitigate these risks, high-fidelity temperature monitoring and precise thermal control are non-negotiable prerequisites for the reliable long-term storage of biospecimens.

Established industry guidelines and national standards provide baseline frameworks for sensor deployed in temperature mapping, but they are inherently limited by their empirical, geometry-based design. The ISPE Good Practice Guide recommends minimum 9 temperature sensors for freezers with a volume up to 2 m^3^ and 15 sensors for units up to 20 m^3^, with placement restricted to geometric corners and central positions [3]. Similarly, the Chinese National Standard GB/T 20154-2014 mandates 5-sensor layout specifically for chest ULT freezers with an inner length over 0.7 m [4]. While these standards ensure basic spatial coverage, they fail to account for the complex thermal dynamics of ultra-large ULT freezers—where heat transfer is governed by a combination of conduction, radiation, and weak natural convection—often resulting in suboptimal sensor placement that overlooks critical thermal hot spots or cold zones. Furthermore, discrete point measurements alone cannot capture the continuous spatial distribution of temperature within the chamber, creating a fundamental need for temperature field reconstruction to achieve a comprehensive understanding of the internal thermal environment. On the other hand, developing a cost-effective optimal sensor placement (OSP) strategy that maximizes reconstruction accuracy with limited sensors has become essential for large-scale ULT freezer monitoring.

In recent years, sparse sensor placement optimization (SSPO) has emerged as a transformative paradigm, enabling high-precision field reconstruction with drastically reduced sensor counts through physics-driven or data-driven frameworks [5,6,7]. Physics-driven approaches leverage fundamental physical laws to define optimization criteria, eliminating reliance on large datasets. For instance, Liu et al. [5] proposed a physics-driven OSP framework for temperature field reconstruction, integrating neural network-based reconstruction models with a genetic algorithm that minimizes the condition number of the physical model matrix. The condition number was identified as a physics-based criterion directly linked to reconstruction error bounds under noisy conditions.

By contrast, data-driven OSP methods capitalize on high-dimensional snapshot data to extract the dominant characteristics of the physical field, with proper orthogonal decomposition (POD) serving as a cornerstone technique for dimensionality reduction and feature extraction. Zhang et al. [6] introduced an entropy-weighted SSPO strategy for ocean surface temperature monitoring, which simultaneously optimizes reconstruction accuracy and information acquisition efficiency. This method innovatively employs temporal entropy to screen high-information training snapshots and spatial entropy to weight POD modes, addressing the information redundancy in large-scale spatiotemporal datasets. QR decomposition with column pivoting (QR-CP) is a widely adopted algorithm for sparse sensor selection in data-driven frameworks [6,8,9]. As demonstrated in Manohar et al. [8], QR pivoting is favorable due to its speed and ease of implementation. Moreover, it inherently optimizes the condition number of the measurement matrix, aligning with D-optimal design principles to enhance numerical stability and noise robustness. In [6], Zhang et al. applied QR-CP to entropy-weighted POD basis to identify the most informative sensor locations efficiently.

The choice of reconstruction model further dictates the performance of data-driven SSPO systems, especially in relation to the modal complexity of the target field. Yuan et al. [10] systematically compared four data-driven reconstruction methods for 2D heat conduction problems: Gappy POD, clustering-based Gappy POD (C-POD), POD-Radial Basis Function (RBF), and POD-Multilayer Perceptron (MLP). Their findings revealed a clear modal dependency: Gappy POD and Gappy C-POD outperformed other methods in low-modal scenarios (1–5 modes), while POD-MLP and POD-RBF excelled in high-modal regimes (above 10 modes). For dynamic systems, Zhou et al. [9] extended 2D temperature field reconstruction for power batteries by integrating a radial basis function (RBF) neural network to predict time-varying POD coefficients, enabling full-state temperature forecasting under sparse sensing conditions.

Despite these advancements, existing SSPO methodologies are rarely tailored to the unique thermal characteristics of ultra-large ULT freezers. To address this research gap, this paper proposes a data-driven optimal sensor placement methodology for efficient temperature monitoring and high-fidelity field reconstruction in large-scale ULT freezers, balancing reconstruction accuracy and cost-effectiveness. The framework proceeds through three core stages. First, prepare high-resolution 3D reference temperature field database. Here, reference fields were obtained through universal kriging (UK) interpolation. A simplified implementation of UK was developed, building on our prior work that introduced adaptive lag-binning and physics-weighted variograms for temperature reconstruction in ULT chest freezer [11]. Second, principal component analysis (PCA) is applied to the training dataset to extract dominant POD modes, establishing a tailored low-dimensional basis that captures the essential thermal dynamics of the ULT freezer. Finally, QR-column pivoting is employed to select the optimal sensor locations from the POD basis. This research establishes a 3D temperature reconstruction framework for ULT freezers in stable operating conditions, which is the operational regime governing long-term biospecimen preservation viability, compliant with GB/T 20154-2014 [4].

This study advances our previous research on kriging-based temperature reconstruction [11] by formalizing a complete OSP pipeline for sparse sensing, with a streamlined universal kriging implementation to generate reliable reference fields for model training and validation.

The remainder of this paper is structured as follows: Section 2 elaborates on the full optimal sensor placement framework, detailing the development of the universal kriging model, the data-driven sensor selection strategy, and the temperature field reconstruction workflow. Section 3 validates the reliability of the kriging-generated reference fields and presents PCA results demonstrating that the first three principal components are sufficient to approximate the full-state temperature field. The reconstruction accuracy achieved with the optimized sensor layout is then quantified and analyzed. Section 4 discusses the key findings of the study, the practical implications of the proposed POD-QR framework for industrial cryogenic storage applications, and its inherent limitations, followed by prospective directions for future research. Finally, Section 5 draws conclusions.

## 2. Materials and Methods

### 2.1. POD-QR Framework for Sparse Sensor Placement Optimization in ULT Freezers

A hybrid methodology for sparse-sensor temperature monitoring was developed, integrating proper orthogonal decomposition and QR-pivoting to balance reconstruction accuracy with sensor economy in cryogenic storage system. The framework comprises seven key steps:

1. Reference field generation: High-resolution temperature fields were established through universal kriging interpolation;
2. Cycle-based data partitioning: Operational cycles were divided into training set (80% of cycles) and testing set (remaining 20%) randomly;
3. POD basis construction: POD basis was extracted from training data, where $\Psi_{r}\in R^{n\times r}$ with rank *r* determined via Kneedle algorithm (singular value distribution and reconstruction error were analyzed);
4. QR-based sensor optimization: Deterministic placement via column-pivoting on $\Psi_{r}$ for target sensor count *p*;
5. Reconstruction validation: Error quantification (RMSE and MAE) using the selected sensor configuration on independent testing data;
6. Parametric analysis: Parametric sweep of *p* (p≥r) through iterative execution of Steps 4–5, the maximum number of sensors was limited to the pre-installed sensors (e.g., 48);
7. Optimal configuration selection: The optimal sensor configuration was determined by applying the Kneedle algorithm to the reconstruction error (RMSE)–sensor count curve.

It is noted that two types of temperature reconstruction methods are introduced in this paper. One is based on universal kriging (UK) interpolation, which was employed to generate high-resolution reference temperature fields. The other is based on POD inverse reconstruction, which was used for temperature field reconstruction with the optimized sparse sensor configuration.

### 2.2. Reference Temperature Fields Construction

Experiments revealed a dominant vertical temperature trend in chest freezers, motivating our use of universal kriging. The high-resolution reference fields were constructed following:

(1) $$T_{\mathrm{ref}}\left(x,y,z\right)=f\left(z\right)+r\left(x,y,z\right),$$

where f(z) represents the vertical polynomial trend and r(x,y,z) the spatial residuals. Prior to UK, the interior space of ULT chest freezer was meshed into a structured grid of 60×40×50 points (X×Y×Z).

The vertical trend was modeled by $L_{1}$-regularized polynomial regression [12]. Bayesian information criterion (BIC) was applied to select the optimal polynomial degree. A lower BIC value indicates a better trade-off between model accuracy and complexity.

Spatial residuals r(x,y,z) underwent 3D kriging interpolation. Leave-one-out cross-validation (LOOCV) identified the optimal variogram from Gaussian, spherical, exponential, and linear candidates. Model performance was evaluated by root mean squared error (RMSE), mean absolute error (MAE) and coefficient of determination ($R^{2}$).

### 2.3. POD Basis Construction

Proper orthogonal decomposition (POD) is widely used in dimensionality reduction tasks [9,10,13]. High-dimensional states $x\in R^{n}$ can be projected into POD subspace, defined by a small number of orthonormal eigenmodes ψ (POD modes) [8]. The full-state x can be represented in low-dimensional form with a linear combination of POD modes and coefficients a:

(2) $$x_{i}\approx \sum_{k=1}^{r}a_{k}\left(t_{i}\right)\psi_{k}\left(x\right).$$

The time series data $x_{i}$ is approximated by a time-varying coefficients $a_{k}\left(t_{i}\right)$ and time-independent spatial modes $\psi_{k}\left(x\right)$.

Let X denote the data matrix

(3) $$X=\left[\begin{array}{cccc} x_{1} & x_{2} & \ldots & x_{m} \end{array}\right]\in R^{n\times m},$$

where *n* is the state dimension and *m* is the sample length.

In our case, the data-matrix was composed of *m* snapshots of reference temperature fields, with *n* grid points (states) in each reference field. Let the *m* snapshots be reshaped into column vectors:

(4) $$X=\left[\begin{array}{cccc} T_{\mathrm{ref}}^{\left(1\right)} & T_{\mathrm{ref}}^{\left(2\right)} & \cdots & T_{\mathrm{ref}}^{\left(m\right)} \end{array}\right]\in R^{n\times m}.$$

Note that row means were subtracted from respective rows prior to POD.

Singular value decomposition (SVD) yields the POD modes:

(5) $$X=\Psi \Sigma V^{T}\approx \Psi_{r}\Sigma_{r}V_{r}^{T},$$

with $\Psi =\left[\psi_{1},\psi_{2},\ldots \psi_{n}\right]\in R^{n\times n}$ containing orthonormal spatial modes. The truncated basis $\Psi_{r}\subset \Psi$, where $\Psi_{r}\in R^{n\times r}$ retains the leading *r* modes determined by satisfying the energy criterion:

(6) $$\frac{\sum_{i=1}^{r}\sigma_{i}^{2}}{\sum_{i=1}^{n}\sigma_{i}^{2}}\geq \eta \;\left(e.g.,\;\;\eta =0.95\right).$$

with $\sigma_{i}$ representing the *i*-th singular value. The POD basis rank *r* was fine tuned with reconstruction accuracy considered.

### 2.4. Sparse Sensor Placement and Reconstruction

Optimal sensor locations for p=r were determined via pivoted QR decomposition [8]:

(7) $$\Psi_{r}^{T}C^{T}=QR,$$

where C denotes the column permutation matrix. Sensor indices $S_{p}=\left\{C_{1},\ldots ,C_{p}\right\}$ correspond to the first *p* pivots. For oversampled cases (p>r), decomposition was applied to $\Psi_{r}\Psi_{r}^{T}$ [8]:

(8) $$\left(\Psi_{r}\Psi_{r}^{T}\right)C^{T}=QR.$$

Here, a modified Gram–Schmidt with column pivoting method was developed to address computational complexity $O(n^{3})$.

Temperature reconstruction combines:

(9) $$\begin{array}{cc} x & \approx \Psi_{r}a\;\left(a\in R^{r}\right), \end{array}$$

(10) $$\begin{array}{cc} y & =Cx\;(y\in R^{p}), \end{array}$$

yielding coefficients $\hat{a}={\left(C\Psi_{r}\right)}^{†}y$, approximated with the Moore–Penrose pseudoinverse (least-square estimation), and reconstructed field:

(11) $$\hat{x}=\Psi_{r}\left\{\begin{array}{cc} {\left(C\Psi_{r}\right)}^{-1}y, & p=r,\\ {\left(C\Psi_{r}\right)}^{†}y, & p>r. \end{array}\right.$$

### 2.5. Experimental Validation

**Figure 1:**
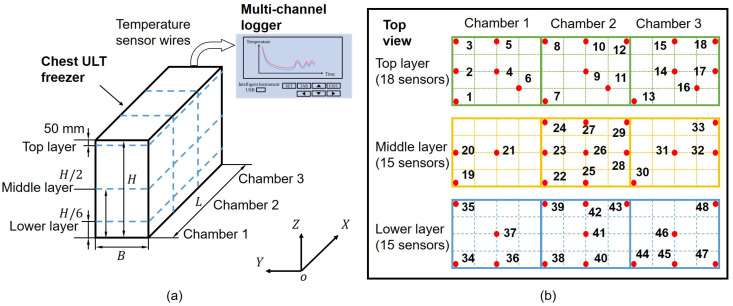
(**a**) Schematic diagram of the experimental setup. Bold black lines denote the interior boundary of the freezer, while dashed blue lines virtually subdivide its internal space. (**b**) Top-view layout showing temperature sensor locations and numbering.

Experimental validations were conducted with two chest ultra-low temperature (ULT) freezers from different manufactures with a cabinet volume of 3 m^3^ (L×B×H=2.47 m×0.905 m×1.385 m). A total of 48 pre-calibrated resistance temperature detectors (RTDs, PT1000, Class B, tolerances ±(0.3+0.005|T|) °C, Shanghai Shenming Automatic Control Instrument Co., Ltd., Shanghai, China) were strategically arranged in three horizontal layers to sample the temperature within the freezers. A 48-channel logger (THW480K, Huikong Technology (Ningbo) Co., Ltd., Ningbo, China) was used to recorder the temperature, see the experimental setup in Figure 1a, with sensor arrangement in Figure 1b. While the national standard [4] specifies upper and lower layers at ^1^/_6_*H* from the top and bottom, respectively, the top layer was intentionally positioned close to the insulation lids (50 mm below) to capture door-induced thermal fluctuations. The middle and lower layers were placed at mid-height and ^1^/_6_*H* from the bottom. In addition to the standard monitoring locations specified in [4], supplementary sensors were placed near the walls and corners to allow a more comprehensive representation of the temperature field.

Experiments were conducted at a controlled environment with a room temperature of 25±1 °C and a relative humidity of 67±4%. The refrigeration cycle was set on at −82 °C and off at −84 °C. The temperature distribution at stable operation phase of the ULT freezers was the focus of the study. Samples were collected at 20 s intervals.

### 2.6. Data Processing

Data processing and analysis were performed using Python 3.12 within PyCharm Community Edition 2024.3.2. The raw data was downsampled, yielding 194 snapshots for Freezer 1 and 191 for Freezer 2. Note that Freezer 1 only had 47 measurements available per snapshot, as one sensor at Location 40 malfunctioned.

The Kneedle algorithm was implemented using the Python kneed library, v0.8.6 without custom threshold adjustments.

## 3. Results

### 3.1. Trend Model

**Figure 2:**
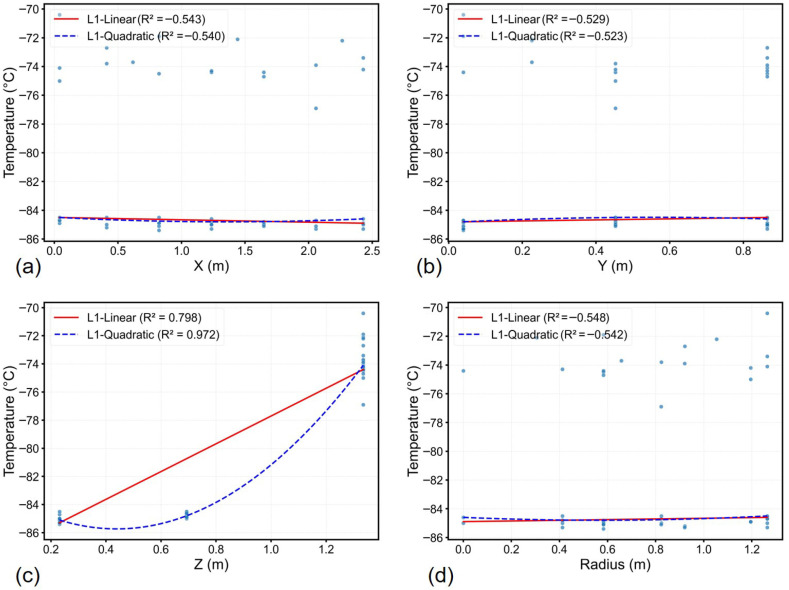
Temperature profile along spatial coordinates with $L_{1}$-norm regression fits: (**a**) X-direction, (**b**) Y-direction, (**c**) Z-direction, (**d**) radial (XY-plane). Dots represent measured temperatures.

The temperature profiles in a ULT chest freezer are presented in Figure 2. Linear regression revealed a strong positive correlation in the Z-direction ($\mathrm{slope}=9.9\;{}^{\circ}Cm^{-1}$, $R^{2}=0.798$), indicating vertical temperature stratification. The trend fitting was further improved with a quadratic regression ($R^{2}=0.972$). In contrast, the negative $R^{2}$ values in the horizontal *X*, *Y* and radial directions reflect temperature uniformity within the horizontal plane.

Comparison of average BIC values for all snapshot data across different polynomial degrees revealed that the second-degree polynomial yielded the minimum BIC value, confirming it as the optimal fitting degree. Specifically, the RMSE decreased from 2.22 °C (first-degree polynomial) to 0.90 °C (second-degree polynomial), while increasing the polynomial degree to three did not result in further RMSE reduction.

### 3.2. Optimal Variogram Models and Interpolation Performance

**Figure 3:**
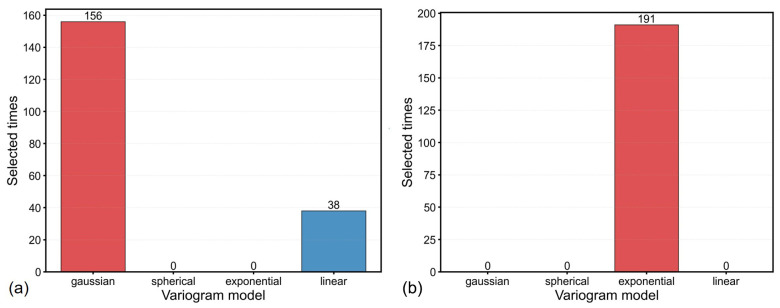
Frequency distribution of selected optimal variogram models across all temperature snapshots. Red bars indicate the best-performing models for (**a**) Freezer 1 and (**b**) Freezer 2.

Cross validation revealed different optimal variogram model for each freezer. Gaussian dominated across Freezer 1 snapshots while exponential model functioned consistently demonstrated superior performance for Freezer 2 (Figure 3). Universal kriging interpolation with optimized parameters achieved high prediction accuracy across both freezers as quantified in Table 1. For Freezer 1, the empirical variogram fitted with a Gaussian model yielded the lowest interpolation errors, with MAE=0.60 °C, RMSE=1.01 °C, and $R^{2}=0.972$. For Freezer 2, the exponential variogram model produced MAE=0.67 °C, RMSE=1.22 °C, and $R^{2}=0.924$. Overall, the optimized UK model enabled construction of high-resolution temperature fields.

### 3.3. Temperature Distribution Analysis

**Figure 4:**
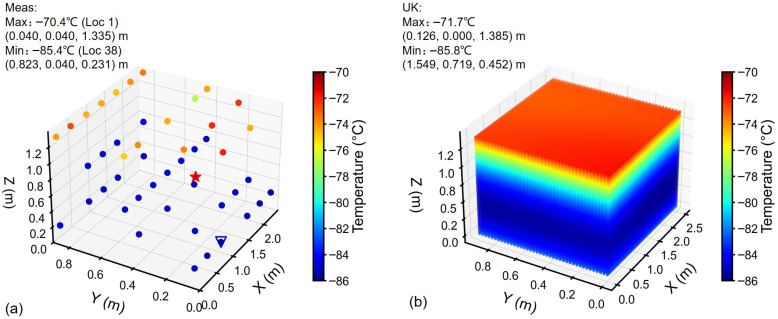
3D temperature distribution in Freezer 1: (**a**) Scatter plot of measured temperature. Red star: hot spot; blue triangular: cold spot. (**b**) Temperature field from universal kriging.

**Figure 5:**
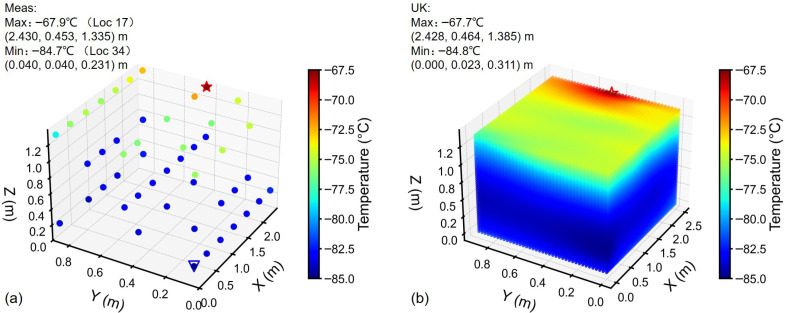
3D temperature distribution in Freezer 2: (**a**) Scatter plot of measured temperature. Red star: hot spot; blue triangular: cold spot. (**b**) Temperature field from universal kriging.

**Figure 6:**
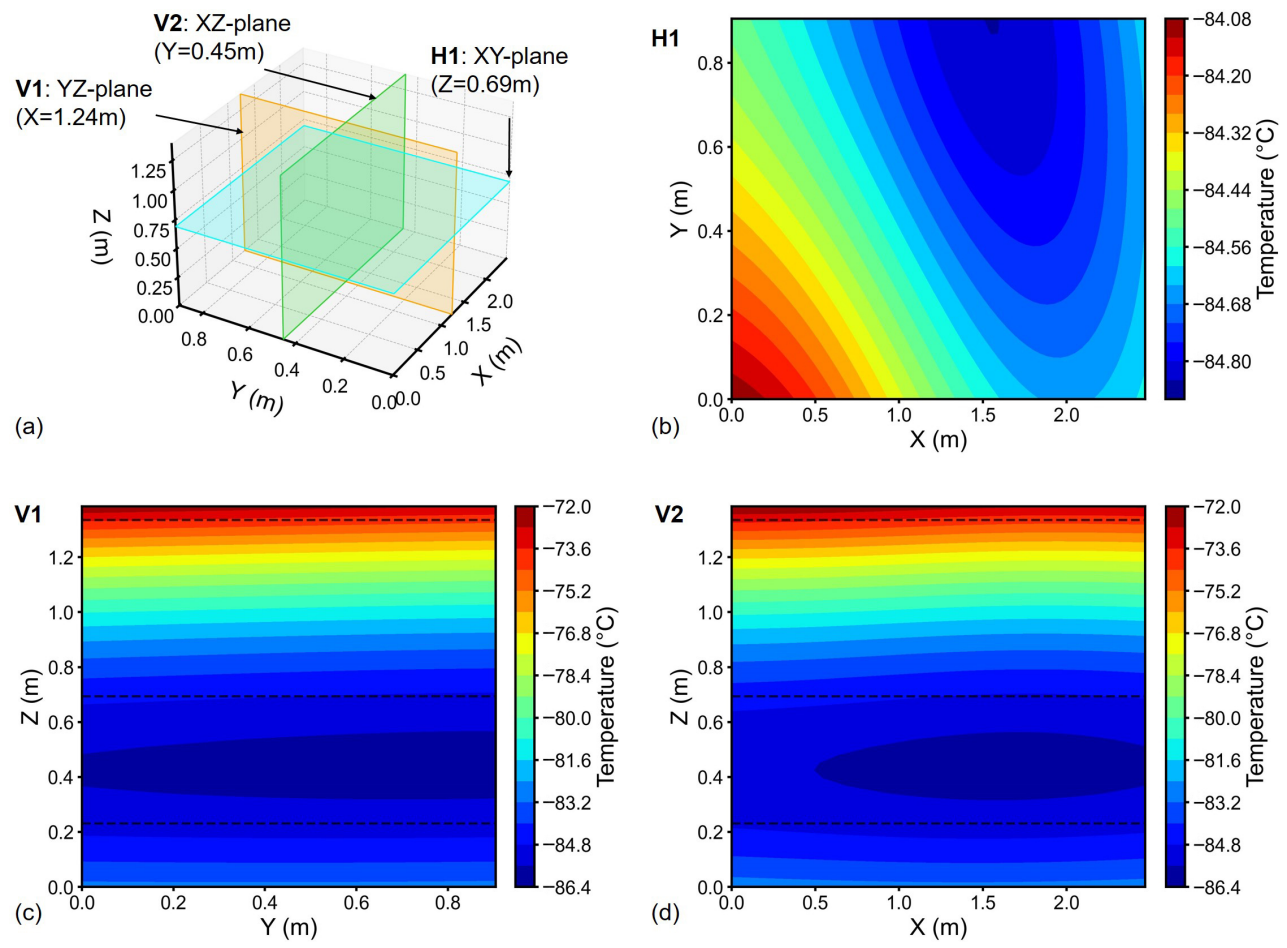
2D temperature cloud maps for Freezer 1: (**a**) Orthogonal reference planes; Interpolated temperatures at (**b**) Z=0.69 m (XY-plane), (**c**) X=1.24 m (YZ-plane), (**d**) Y=0.45 m (XZ-plane). Black dashed lines: sensors heights (50 mm below top lid, ^1^/_2_*H* and ^1^/_6_*H* from bottom).

**Figure 7:**
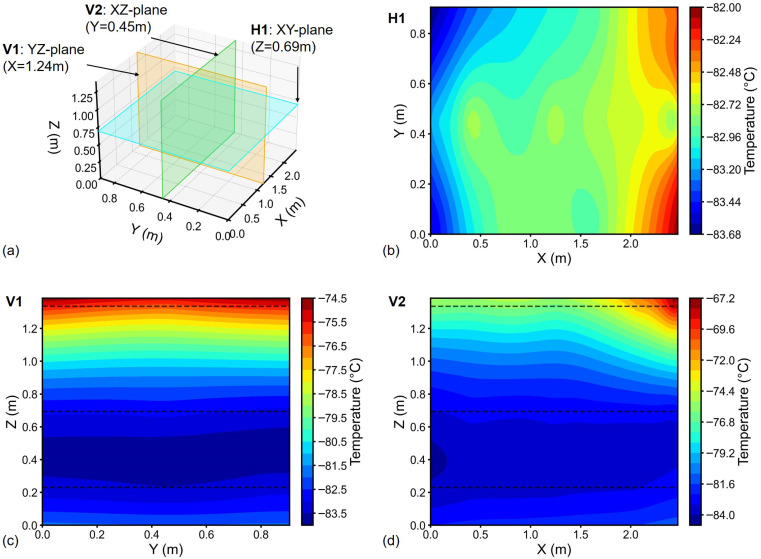
2D temperature cloud maps for Freezer 2: (**a**) Orthogonal reference planes; Interpolated temperatures at (**b**) Z=0.69 m (XY-plane), (**c**) X=1.24 m (YZ-plane), (**d**) Y=0.45 m (XZ-plane). Black dashed lines: sensors heights (50 mm below top lid, ^1^/_2_*H* and ^1^/_6_*H* from bottom).

Temperature distribution was analyzed considering the integral mean temperature at each measurement point. From measurements of Freezer 1, hot/cold spots were identified at Location (Loc) 1 (top layer) and at Loc 38 (lower layer, 0.231 m height), respectively (Figure 4a). The temperature field via UK interpolation Figure 4b positioned the cold spot at approximately 0.452 m height (approximately two times higher than Loc 38). The hot spot was near the top-left corner, adjacent to Loc 1. In case of Freezer 2, the hot spot was identified at Loc 17 (top layer) and cold spot at Loc 34 (lower layer), see Figure 5a. The hot spot location obtained through interpolation and that obtained through measurement were almost identical (Figure 5b). While the cold spot was found at a height above the lower sensing layer (^1^/_6H_). The hot/cold spots can also refer to the 2D temperature cloud maps in Figure 6 and Figure 7.

Interpolated temperature distributions in three orthogonal central planes of Freezer 1 are shown in Figure 6. Horizontal plane H1 at Z=0.69 m (Figure 6b) exhibited temperature uniformity ($\Delta T_{H1}<1\;{}^{\circ}C$). Conversely, vertical cross-sections V1 and V2 (Figure 6c,d) displayed pronounced vertical temperature gradients ($|\nabla T_{z}|>5.0\;{}^{\circ}C/m$), indicating vertically dominant thermal variation pattern within the chamber. Similar thermal patterns were obtained for Freezer 2 (Figure 7). In Freezer 2, the Loc 17 hot spot significantly influenced V2 plane thermal distribution (Figure 7d).

### 3.4. Principal Component Analysis

**Figure 8:**
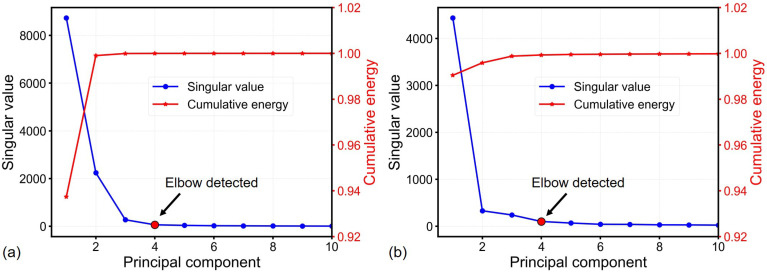
Singular values and cumulative energy of the first 10 principal components in training data: (**a**) Freezer 1 and (**b**) Freezer 2. Red dots indicate elbows detected by the Kneedle algorithm.

Figure 8 shows the singular values and cumulative energy distribution of the first 10 principal components for both freezer units. The Kneedle algorithm identified four dominant modes through scree plot of singular values. Table 2 summarizes the cumulative energy contributions of these primary components. Energy capture exceeding 99.8% in both units by first three modes highlights the low-rank structure of temperature fields.

**Figure 9:**
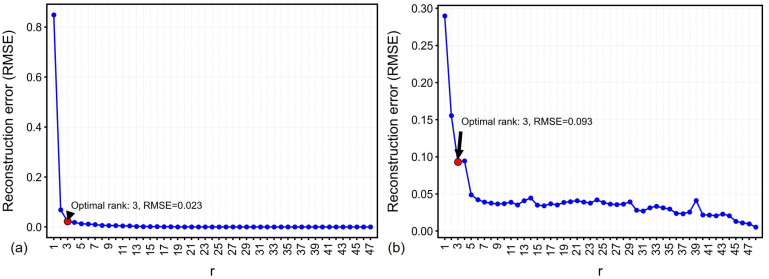
Reconstruction error versus POD basis rank *r* (let p=r) in testing data: (**a**) Freezer 1. (**b**) Freezer 2. Red dots indicate optimal ranks.

On the other hand, reconstruction performance dependence on POD basis rank *r* (with sensor count p=r) is quantified in Figure 9. The Kneedle algorithm applied to RMSE profiles determined r=3 as the optimal basis rank for both units, achieved overall RMSE less than 0.1 °C. This validates that the full-state temperature fields can be successfully reconstructed using the low-rank POD basis (r=3). These optimized rank-3 bases were retained for downstream QR decomposition given their mathematically compact representation.

### 3.5. Optimal Sensor Placement via QR-Pivoting and Reconstruction Performance

**Figure 10:**
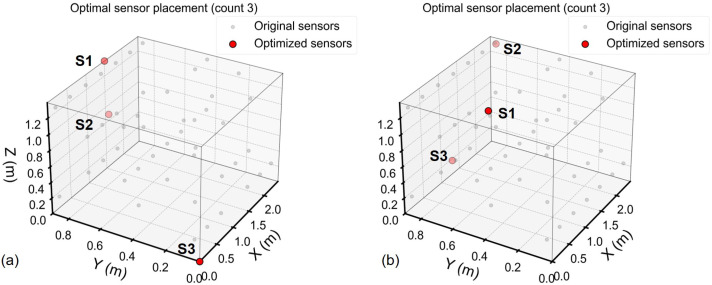
Optimal sensor placements identified by QR-pivoting: (**a**) Freezer 1. (**b**) Freezer 2. Red dots mark selected sensors (S1–S3).

The optimal sensor locations were selected using the tailored POD basis (r=3) and QR-pivoting. Reconstruction accuracy was evaluated across sensor counts ranging from the optimal rank to the full 48-sensor experimental configuration. For each freezer, the sparse sensor optimization approach converged to p=3 as the optimal sensor count. The resulting sensor placements are visualized in Figure 10, with coordinate specifications provided in Table 3.

The optimized configurations achieved:

- Grid-level accuracy: Sub-0.1 °C errors relative to reference temperature (Table 4)
- Point-level validation against experimental measurements:
  – Freezer 1: MAE = 0.502 °C, RMSE = 0.842 °C
  – Freezer 2: MAE = 0.138 °C, RMSE = 0.167 °C

**Figure 11:**
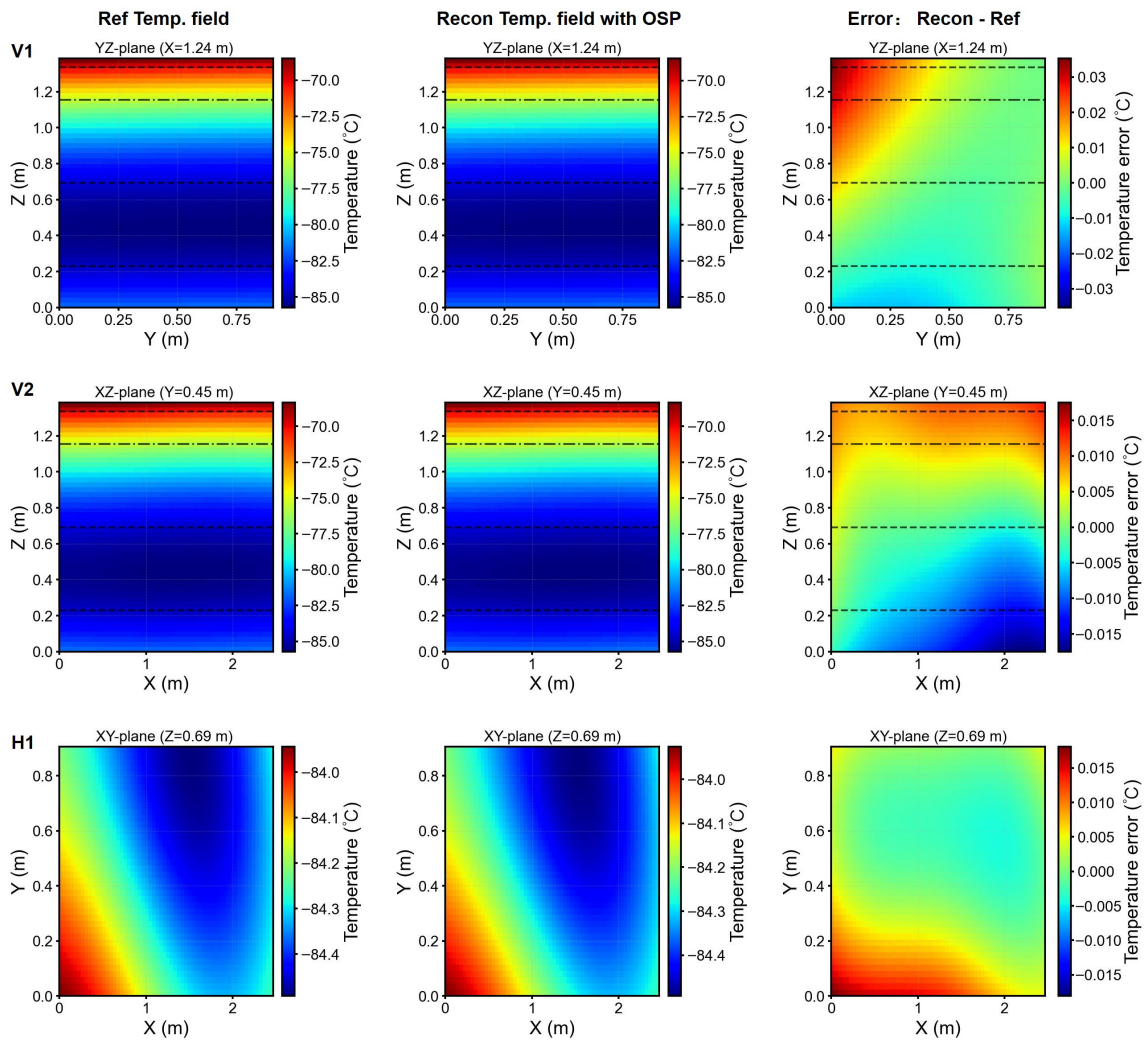
Freezer 1 reconstruction: orthogonal vertical planes (V1, V2) and horizontal plane (H1). Columns: reference fields (**left**), optimal-sensor based reconstruction (**center**), and error distribution (**right**). Black dashed lines: sensors heights; black dash-dotted line: ^1^/_6_*H* below the top lid.

**Figure 12:**
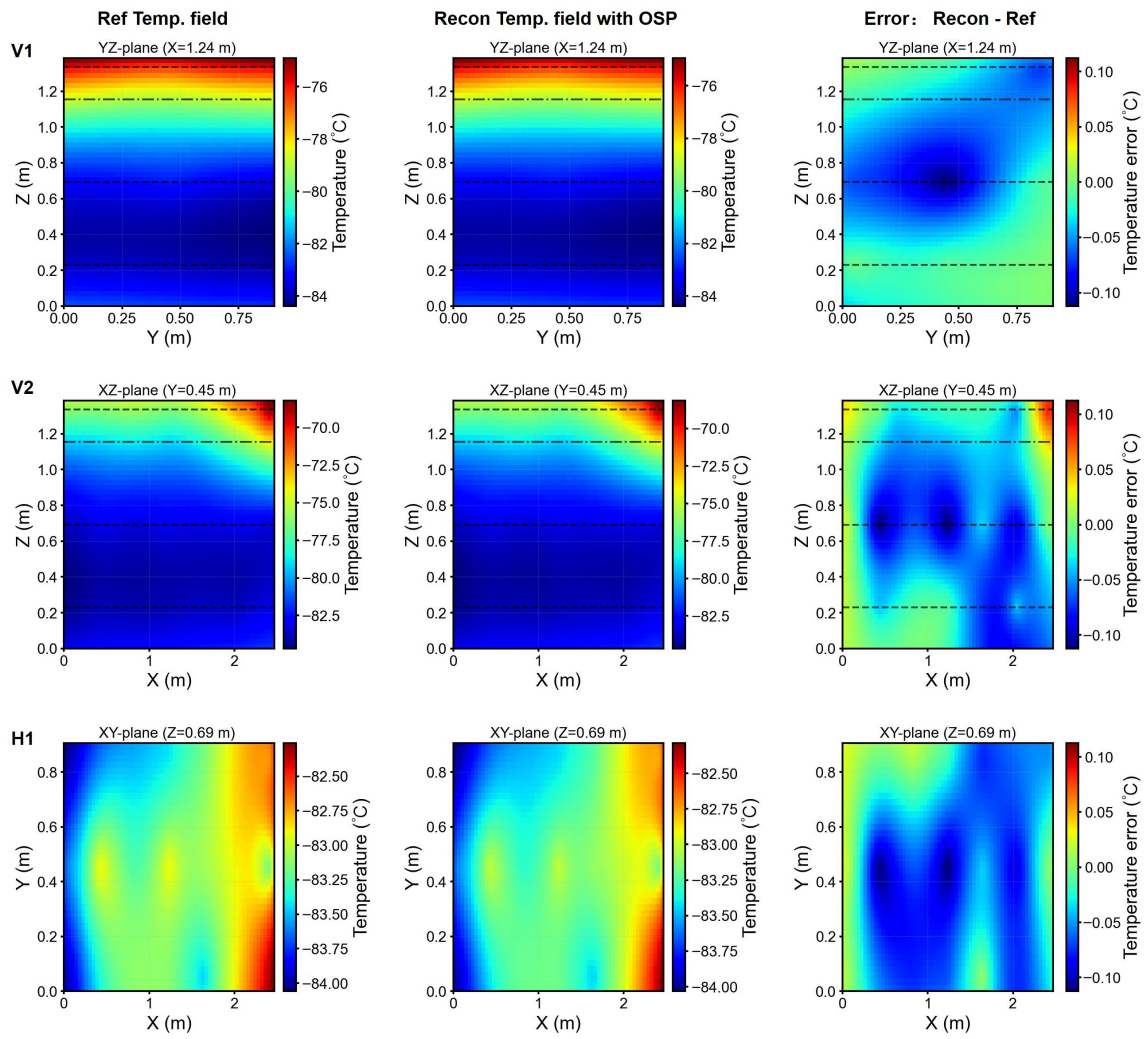
Freezer 2 reconstruction: orthogonal vertical planes (V1, V2) and horizontal plane (H1). Columns: reference fields (**left**), optimal-sensor based reconstruction (**center**), and error distribution (**right**). Black dashed lines: sensors heights; black dash-dotted line: ^1^/_6_*H* below the top lid.

Reconstruction fidelity remained high for all cases, with a coefficient determination $R^{2}>0.97$. This outcome achieves a 94% reduction in sensor requirements (from 48 to 3 sensors) while preserving predictive accuracy. Figure 11 (Freezer 1) and Figure 12 (Freezer 2) present temperature field reconstructions and error distributions for representative test scenarios employing the optimal sensor placement (p=3). The low-rank model effectively captured key thermal characteristics of the freezers, including hot spots and vertical temperature gradients.

## 4. Discussion

In general, the temperature distribution in ULT chest freezers is uniform horizontally, with dominant variations occurring along the vertical (height) direction. Notably, quadratic regression ($R^{2}=0.972$) outperformed linear fitting (Figure 2c) to the vertical trend, and the Bayesian Information Criterion confirmed the second-degree polynomial as the optimal trend model. Hence, in temperature mapping of ULT freezer, focusing on vertical coverage is critical to capturing meaningful thermal variation. For example, temperature fields identified the cold spots between ^1^/_2_*H* and ^1^/_6_*H* (Figure 6 and Figure 7), which encourages the sensor placement at height of ^1^/_3_*H* in addition to the three sensor layers.

The optimized UK model demonstrated robust performance in reconstructing high-fidelity temperature fields. In terms of variogram model dependence, Freezer 1 consistently favored the Gaussian variogram model, while Freezer 2 performed best with the exponential model (Figure 3). Gaussian models are typically associated with smooth, continuous spatial variations (e.g., gradual vertical stratification), while exponential models better capture abrupt transitions (e.g., localized heat leakage near sensor wire penetrations in Freezer 2). The UK framework’s ability to adapt to such unit-specific thermal dynamics highlights its flexibility for real-world applications. Interpolation accuracy evaluated by LOOCV (Table 1) showed high predictive power for both freezers: Freezer 1 achieved MAE=0.60 °C, RMSE=1.01 °C, and $R^{2}=0.972$, while Freezer 2 yielded MAE=0.67 °C, RMSE=1.22 °C, and $R^{2}=0.924$.

It is crucial to clarify that the observed differences in thermal characteristics between the two freezers—including distinct optimal variogram models (Gaussian vs. exponential), divergent hotspot locations (Loc 1 vs. Loc 17), and consequent variations in optimal sensor positions—may be attributed to the combined effect of two key factors. First, the freezers are from different manufacturers, with notable discrepancies in evaporator design (e.g., layout, heat exchange area, and airflow guidance structure) that directly shape internal cold flow distribution and thermal gradient patterns. Second, during experiments, the wiring terminals of the 48 sensors were routed out beneath the top door (adjacent to one side wall along the depth direction), with wire exit positions sealed using dedicated materials to prevent thermal transfer with the external environment. However, subtle variations in the manual sealing process, coupled with manufacturer-specific structural differences at the top door-side wall junction, may have led to slight inconsistencies in sealing effectiveness between the two units, resulting in localized heat leakage to varying degrees. These combined influences disrupted the thermal field differently for each freezer, ultimately causing the observed variations in thermal characteristics and optimal sensor configurations. This underscores the necessity of unit-specific optimization for sensor placement in low-temperature storage systems.

Principal component analysis uncovered a highly compact, low-rank representation of the temperature fields, with the first three principal modes capturing >99.7% of the cumulative energy for both freezers (Table 2). This low modal complexity aligns with the simplified thermal dynamics of ULT freezers—dominated by vertical stratification and a small number of localized thermal anomalies (e.g., hot spots near door seals). For context, a parallel study on 2D temperature distribution in power batteries also demonstrated that the first three modes captured 99.68% of system energy [9], enabling effective temperature reconstruction via a reduced-order model. The Kneedle algorithm confirmed r=3 as the optimal POD basis rank, with reconstruction errors less than 0.1 °C, further validating that the full-state temperature field can be accurately approximated using a minimal set of dominant modes. Notably, the low modal complexity inherent to ULT freezers lays the foundation for drastic sensor reduction in sparse sensing frameworks.

The QR-pivoting algorithm was applied to the optimized rank-3 POD basis to identify the optimal sensor placement with a target sensor count *p*. A parametric sweep of *p* revealed an optimal sensor count of 3 for both freezers, representing a 94% reduction in sensor requirements (from 48 to 3) while preserving exceptional reconstruction fidelity. Key performance outcomes include: grid-level accuracy shows reconstruction errors below 0.1 °C and $R^{2}>0.999$, confirming near-perfect alignment with reference fields (Table 4); point-level validation yields MAE values of 0.502 °C (Freezer 1) and 0.138 °C (Freezer 2) against experimental measurements, with $R^{2}>0.97$ for both units. It is acknowledged that the 48 validation points may not fully capture all potential error regions of the UK model; however, the consistent high accuracy across both grid-level and point-level comparisons supports the practical adequacy of the approach. While QR pivoting is theoretically linked to improved noise robustness [8], the present study did not empirically quantify the effect of sensor noise on reconstruction accuracy. Future work should include noise injection experiments to validate this property.

Despite the satisfactory performance in quasi-static temperature field reconstruction for chest ultra-low temperature freezers, the proposed POD–QR framework has several limitations worth noting. First, the method relies on sufficient high-quality reference snapshots: insufficient training data or excessive measurement noise may degrade the accuracy of POD basis extraction and subsequent optimal sensor placement. Thus, preprocessing steps such as outlier removal and data normalization are recommended to improve reliability and stability. Second, as a linear dimensionality reduction technique, POD is inherently constrained in characterizing strong nonlinear thermal behaviors (e.g., sharp temperature gradients, rapid transient fluctuations, or local abrupt thermal anomalies), which may reduce reconstruction fidelity under extreme operating conditions. Third, the optimized POD modes and sensor locations are case-specific and sensitive to physical boundary conditions, including door opening events, internal loading configurations, cabinet geometry, and refrigeration control strategies. Consequently, the obtained sensor configuration may require recalibration when applied to different freezer models or varying working conditions.

Notably, these limitations are manageable within the practical context of cryopreservation applications, where sample integrity is the paramount priority and the value of biological specimens substantially outweighs calibration costs. Consequently, comprehensive temperature calibration constitutes an essential quality assurance measure. This aligns with industry standards requiring all ULT freezers to undergo rigorous temperature mapping during factory acceptance testing prior to deployment. To enhance methodological scalability, we have standardized data acquisition protocols and developed modular test fixtures adaptable across freezer configurations. While this work constitutes a pilot study, systematic expansion to multiple units of the same model and diverse freezer models from different manufacturers will enable derivation of generalized sensor placement guidelines. Critically, even with optimized generalized placements, high-density calibration remains mandatory to: validate unit-specific thermal behaviors during initial commissioning, establish baseline performance metrics, and comply with regulatory requirements for critical storage infrastructure. Importantly, such resource-intensive calibration need not be continuous; it can be implemented as periodic validation (e.g., quarterly/bi-annually) after establishing baseline performance through the initial full-grid characterization.

While the current work focuses on temperature reconstruction during the stable operating conditions of ULT freezers, future work will extend the methodology to transient thermal behaviors (e.g., door openings, cooling system failures) where modal complexity temporarily increases. Dynamic mode decomposition (DMD) is well-suited for transient analysis, as it efficiently extracts spatio-temporal dynamic modes with associated frequencies and growth/decay rates [14]. For nonlinear thermal patterns, CNN-based autoencoder method, such as the attention-augmented reconstruction framework proposed by Khan et al. [15], was powerful for high-complexity 3D temperature fields (e.g., fuel plant combustion environments with 0 °C to 850 °C operating ranges, significant temperature gradients and nonlinearities). The superiority of our POD-based approach lies in its alignment with the low-modal, quasi-static thermal dynamics of ULT freezes. While DMD excels at capturing transient dynamics [14] and autoencoders [15] at nonlinear, high-complexity temperature distributions, our framework is tailored to the practical demands of cryogenic storage: minimal sensor count, physical interpretability of modes, and low computational overhead. We acknowledge the complementary potential of DMD and autoencoders for extended scenarios: DMD for transient events where temporal dynamics are prominent (e.g., door openings); autoencoders for ULT freezers with complex thermal anomalies. We plan to explore these hybrid approaches in future work.

A second key direction for future research is enhancing the energy efficiency of cryogenic storage systems: strategically placed sensors are anticipated to reduce energy consumption while maintaining target temperature setpoints. A recent study demonstrated a 19.8% reduction in energy consumption for cold storage environments (target temperature: −10 °C) using a continuous Deep Deterministic Policy Gradient (DDPG)-based control algorithm [16], validating the potential of sensor-driven energy optimization for cryogenic applications.

## 5. Conclusions

Reliable temperature mapping in ultra-low temperature (ULT) chest freezers is essential for preserving biospecimen integrity in cryogenic biobanking, yet it typically demands a dense array of sensors to ensure accuracy. In practical applications, however, sparse sensor deployment is often unavoidable due to constraints including limited installation space and cost limitations. These situations present a critical challenge: achieving precise temperature field characterization in ULT freezers using a minimal number of sensors. To address this challenge, a systematic data-driven framework for optimal sparse sensor placement is proposed, enabling high-precision 3D temperature field reconstruction while reconciling the trade-off between monitoring accuracy, spatial feasibility, and cost-effectiveness.

Experimental validation on two large-scale ULT chest freezers confirmed the effectiveness and robustness of the proposed framework under stable operating conditions. Temperature fields have a low-rank structure, with 3 POD modes capturing over 99.8% of cumulative energy, laying the foundation for sparse sensor deployment. Leveraging this low-rank feature, QR-pivoting optimization enabled a 94% reduction in sensor requirements, from 48 full-scale sensors to a minimal configuration of 3, while maintaining sub-0.1 °C grid-level reconstruction accuracy and strong agreement with experimental measurements ($R^{2}>0.97$). This work offers a viable, data-driven solution for thermal management in large-scale ULT storage systems.

The proposed framework advances sparse sensing methodologies for cryogenic thermal monitoring, supporting the reliable preservation of biospecimens critical to biobanking, clinical research, and public health initiatives.

## Figures and Tables

**Table 1:** Universal kriging parameters and aggregated LOOCV performance metrics.

Case	Snapshots	Trend Degree/Variogram Model	MAE (°C)	RMSE (°C)	$R^{2}$
Freezer 1	194	2/Gaussian	0.60	1.01	0.972
Freezer 2	191	2/Exponential	0.67	1.22	0.924

**Table 2:** Cumulative energy of the dominant POD modes.

POD Mode	Cumulative Energy (Freezer 1)	Cumulative Energy (Freezer 2)
1	93.74%	99.04%
2	99.90%	99.59%
3	99.99%	99.88%
4	100%	99.93%

**Table 3:** Spatial coordinates (unit: m) of optimal sensors.

	Freezer 1			Freezer 2		
Sensor	X	Y	Z	X	Y	Z
S1	1.507	0.905	1.385	0.419	0.464	1.385
S2	1.591	0.905	0.678	2.428	0.859	1.328
S3	0	0	0	1.214	0.882	0.226

**Table 4:** Reconstruction performance of optimized sensor networks.

Case	Sensor Count	Grid Level			Point Level		
		MAE (°C)	RMSE (°C)	*R* ^2^	MAE (°C)	RMSE (°C)	*R* ^2^
Freezer 1	3	0.018	0.023	1.000	0.502	0.842	0.971
Freezer 2	3	0.079	0.093	0.999	0.138	0.167	0.998

## Data Availability

The original contributions presented in this study are included in the article. Further inquiries can be directed to the corresponding author.

## Abbreviations

The following abbreviations are used in this manuscript:

LOOCV	Leave-one-out cross-validation
OSP	Optimal sensor placement
PCA	Principal component analysis
POD	Proper orthogonal decomposition
SSPO	Sparse sensor placement optimization
UK	Universal kriging
ULT	Ultra-low temperature freezer
MAE	Mean absolute error
RMSE	Root mean squared error
DMD	Dynamic mode decomposition
